# Identification of COVID-19 severity biomarkers based on feature selection on single-cell RNA-Seq data of CD8^+^ T cells

**DOI:** 10.3389/fgene.2022.1053772

**Published:** 2022-11-09

**Authors:** Jian Lu, Mei Meng, XianChao Zhou, Shijian Ding, KaiYan Feng, Zhenbing Zeng, Tao Huang, Yu-Dong Cai

**Affiliations:** ^1^ Department of Mathematics, School of Sciences, Shanghai University, Shanghai, China; ^2^ CAS Key Laboratory of Computational Biology, Bio-Med Big Data Center, Shanghai Institute of Nutrition and Health, University of Chinese Academy of Sciences, Chinese Academy of Science, Shanghai, China; ^3^ State Key Laboratory of Oncogenes and Related Genes, Center for Single-Cell Omics, School of Public Health, Shanghai Jiao Tong University School of Medicine, Shanghai, China; ^4^ School of Life Sciences, Shanghai University, Shanghai, China; ^5^ Department of Computer Science, Guangdong AIB Polytechnic College, Guangzhou, China; ^6^ CAS Key Laboratory of Tissue Microenvironment and Tumor, Shanghai Institute of Nutrition and Health, University of Chinese Academy of Sciences, Chinese Academy of Sciences, Shanghai, China

**Keywords:** COVID-19, severity, CD8^+^ T cell, single-cell, feature selection

## Abstract

The global outbreak of the COVID-19 epidemic has become a major public health problem. COVID-19 virus infection triggers a complex immune response. CD8^+^ T cells, in particular, play an essential role in controlling the severity of the disease. However, the mechanism of the regulatory role of CD8^+^ T cells on COVID-19 remains poorly investigated. In this study, single-cell gene expression profiles from three CD8^+^ T cell subtypes (effector, memory, and naive T cells) were downloaded. Each cell subtype included three disease states, namely, acute COVID-19, convalescent COVID-19, and unexposed individuals. The profiles on each cell subtype were individually analyzed in the same way. Irrelevant features in the profiles were first excluded by the Boruta method. The remaining features for each CD8^+^ T cells subtype were further analyzed by Max-Relevance and Min-Redundancy, Monte Carlo feature selection, and light gradient boosting machine methods to obtain three feature lists. These lists were then brought into the incremental feature selection method to determine the optimal features for each cell subtype. Their corresponding genes may be latent biomarkers to determine COVID-19 severity. Genes, such as ZFP36, DUSP1, TCR, and IL7R, can be confirmed to play an immune regulatory role in COVID-19 infection and recovery. The results of functional enrichment analysis revealed that these important genes may be associated with immune functions, such as response to cAMP, response to virus, T cell receptor complex, T cell activation, and T cell differentiation. This study further set up different gene expression pattens, represented by classification rules, on three states of COVID-19 and constructed several efficient classifiers to distinguish COVID-19 severity. The findings of this study provided new insights into the biological processes of CD8^+^ T cells in regulating the immune response.

## 1 Introduction

Caused by severe acute respiratory syndrome coronavirus 2 (SARS-CoV-2), coronavirus disease 2019 (COVID-19) has cumulatively infected more than 400 million people. It is mainly transmitted in the population through close contact, and typical clinical symptoms are fever and cough (Sanyal, 2020). SARS-CoV-2 enters host cells through endocytosis by binding to angiotensin-converting enzyme 2 (ACE2) receptor on the cell surface (Samudrala et al., 2020). Several variants have emerged, and the main ones are Alpha, Beta, Gamma, Delta, Lambda, and Omicron (Araf et al., 2022; Fiolet et al., 2022).

Viral infections involve a complex immune response process, in which T lymphocytes, especially CD8^+^ T cells, are crucial to the control and clearance of acute infections. CD8^+^ T lymphocytes can selectively kill infected cells by mediating adaptive cytotoxic T cell responses, thereby eliminating the virus (Westmeier et al., 2020). CD8^+^ T cells exert cytotoxic effects mainly through target cell lysis and cytokine release (Slifka and Whitton, 2000). In the target cell lysis pathway, target cells are lysed through the Fas/FasL pathway or perforin, whereas the cytokine pathway is associated with IFNγ and TNFα. Strong CD8^+^ T cell responses specific to SARS-CoV-2 are associated with worse disease severity; SARS-CoV-2 infection results in a decrease in CD8^+^ T cell frequency, which becomes more pronounced with increasing infection severity (Chen et al., 2020). SARS-CoV-2-specific CD8^+^ T-cell responses are rarely detected in patients with fatal COVID-19 (Dan et al., 2021) because of CD8^+^ T-cell depletion after overactivation, which ultimately reduces the host cellular immune response to the virus (Zheng et al., 2020; Gong et al., 2021).

Cellular immunity involves the transformation of naive, effector, and memory T cells. The proportion of CD8^+^ T cell subsets correlates with COVID-19 severity (Westmeier et al., 2020). Patients with moderate COVID-19 have a significantly increased proportion of effector CD8^+^ T cells and effector memory CD8^+^ T cells than healthy subjects and severely infected patients (Fenoglio et al., 2021), whereas naïve CD8^+^ T cells are reduced in old people and negatively correlated with patient age (Westmeier et al., 2020). Naïve CD8^+^ T cells correlated with age and differed across infection status (unexposed, acute, and recovering patients) (Grifoni et al., 2020). In contrast, studies between groups of COVID-19 patients showed that those with severe infection exhibited higher levels of naive CD8^+^ T cells and lower levels of effector CD8^+^ T cells and effector memory CD8^+^ T cells compared with patients with mild infection (Fenoglio et al., 2021), which may imply a defective cytotoxic lymphocyte response in severe infections. In addition, in COVID-19 patients, the dominant effector CD8^+^ T cells were GzmA, GzmB, and perforin triple-positive cells, compared with uninfected individuals; patients expressing effector CD8^+^ T cells that produce multiple virulence molecules exhibited milder symptoms (Westmeier et al., 2020), which may indicate a potential protective mechanism.

As the viral infection subsides, some T cells differentiate into memory T cells. Memory T cells can persist in patients for long periods of time; thus, they play a protective role in preventing viral reinfection (Nguyen et al., 2019). Compared with non-hospitalized patients, hospitalized patients did not have a higher frequency of memory CD8^+^ T cells, and the proportion tended to be stable over time (Grifoni et al., 2020). SARS-CoV-2-specific memory CD8^+^ T cells were related to less severe COVID-19 during infection, because SARS-CoV-2 memory T cells can limit the accumulation of SARS- CoV-2 and viral load, thereby reducing COVID-19 disease severity (Kotturi et al., 2007; Francis et al., 2022). As CD8^+^ T cells are crucial to the infection of SARS-CoV-2, studying the characteristics of different types of CD8^+^ T cells in different infection states provides a useful reference for finding potential targets for treatment.

In this study, several computational methods were used to investigate the gene expression profiles of three subtypes of CD8^+^ T cells (effector, memory and naïve T cells) related to COVID-19. Three disease states: unexposed, acute, and convalescent, were included in the profiles on each cell subtype. The profiles on each cell subtype were individually analyzed in the same way. First, the profiles were analyzed by Boruta feature selection method (Kursa and Rudnicki, 2010) to exclude irrelevant gene features. Then, three feature ranking algorithms: Max-Relevance and Min-Redundancy (mRMR) (Peng et al., 2005), Monte Carlo feature selection (MCFS) (Draminski et al., 2008), and light gradient boosting machine (LightGBM) (Ke et al., 2017), were used to examine remaining features, resulting in three feature lists. Each list was fed into the incremental feature selection (IFS) method (Liu and Setiono, 1998) to extract essential gene features, construct efficient classifiers and set up classification rules. The essential genes can be latent biomarkers and the rules can indicate different expression patterns on three COVID-19 states, deepening our understanding on COVID-19.

## 2 Materials and methods

### 2.1 Datasets

The gene expression profiles of three subtypes of CD8^+^ T cells related to COVID-19, including effector, memory, and naïve T cells, were obtained from the GEO database by accessing a number of GSE188429 (Francis et al., 2022). These expression profiles were obtained by isolating CD8^+^ T cells from individual peripheral blood mononuclear cells (PBMCs) and quantifying mRNA expression in the cells by single-cell transcriptome sequencing techniques. CD8^+^ T cell responses in PBMCs from three cohorts were studied, as follows: acute COVID-19, convalescent COVID-19, and unexposed individuals. A total of 145,293 cell samples were included in these profiles and the number of samples under different cohorts for each CD8^+^ T cell subtype is shown in Table 1. After filtering low expression and low variance genes, 1046 genes were kept and deemed as features in this study. We used the processed data in h5ad file acquired from https://www.ncbi.nlm.nih.gov/geo/query/acc.cgi?acc=GSE188429 for detailed analysis. For the next round of machine learning computations, the datasets for each of the three different cell subtypes were studied independently.

**TABLE 1:** Sample size for the different categories under the datasets for three cell subtypes.

Cell subtype	Acute COVID-19	Convalescent COVID-19	Unexposed individuals	Total
Effector T cells	4832	23542	21288	49662
Memory T cells	6527	22031	28257	56815
Naïve T cells	5204	21504	12108	38816

### 2.2 Boruta feature selection

Lots of gene features were used to represent each cell in three subtypes of CD8^+^ T cells. Evidently, only a few of them are highly related to distinguish the states of COVID-19. It is essential to discover them. This task can be completed by some feature analysis methods. Here, the Boruta feature selection method (Kursa and Rudnicki, 2010) was adopted first to exclude irrelevant features.

The Boruta feature selection method is a feature selection wrapper algorithm, which can be used to assess the importance of features using a tree classifier (e.g., random forest (RF) (Breiman, 2001)) and hence reject irrelevant features. The approach particularly creates a shadow feature at random for each original feature and then compares them with the original features in terms of their importance generated by RF. An original feature is selected when it is statistically more important than the shadow features. Selected features are removed from the current dataset and the dataset containing remaining features is processed in the next round. Above procedures repeat several times until the number of rounds reaches the predefined value.

The present study used the Boruta program available at https://github.com/scikit-learn-contrib/boruta_py to analyze the datasets individually for three cell subtypes. It was run with default parameters.

### 2.3 Feature ranking methods

Important features can be extracted through Boruta. However, their importance was not clear. Three feature analysis methods followed to investigate selected features, including mRMR (Peng et al., 2005), MCFS (Draminski et al., 2008) and LightGBM (Ke et al., 2017).

#### 2.3.1 mRMR

The mRMR uses mutual information as a metric to achieve the maximum correlation between features and class labels as well as the minimum redundancy between features. After mRMR analysis, features are ranked in a list. The list is produced by repeatedly selecting a feature with maximum correlation to class labels and minimum redundancy to already-selected features. For convenience, this list was called the mRMR feature list.

#### 2.3.2 MCFS

The MCFS method is another effective feature selection method in machine learning. The method evaluates the importance of features by constructing a number of decision trees. Trees are set up on some randomly generated feature groups and sample sets. According to the occurrence of each feature in all trees, a relative importance (RI) score is computed and assigned to the feature to indicate its importance. With the decreasing order of RI scores, features are ranked in a list, named MCFS feature list.

#### 2.3.3 LightGBM

The LightGBM represents ensemble learning algorithms and is a distributed gradient-boosting framework based on decision tree algorithm. As the algorithm is based on a tree classifier, it can be used to evaluate the importance of a feature by counting its frequency in all trees. Likewise, features are ranked in a list with the decreasing order of their frequencies. Such list was termed as LightGBM feature list.

In this investigation, the mRMR program used is obtained from http://home.penglab.com/proj/mRMR/. As for the MCFS program, the software developed by Draminski et al. (2008) was adopted, which can be accessed at http://www.ipipan.eu/staff/m.draminski/mcfs.html. The LightGBM program was implemented using the LightGBM library in python, which is available at https://lightgbm.readthedocs.io/en/latest/. The default parameters were used in all above three programs.

### 2.4 Incremental feature selection

After feature ranking, three feature lists for one subtype of CD8^+^ T cells were obtained. However, it was not easy to determine the optimal features from these feature lists. In this step, the IFS method (Liu and Setiono, 1998) was employed to determine the optimal features in each list for a given classification algorithm. The procedures were described as below. When the step size was set to 1, the IFS method first generated a succession of feature subsets in a way that the first feature subset contained the first feature in the list, and the second feature subset included the top two features, and so on. For each feature subset, a classifier was built based on samples represented by features in this subset. All classifiers’ performance was tested using 10-fold cross-validation (Kohavi, 1995). Finally, based on the performance indicators of each classifier, the classifier with the best performance can be obtained. Such classifier was called the optimal classifier and features used in this classifier were termed as the optimal features.

### 2.5 SMOTE

As shown in Table 1, the sizes of different categories in the gene expression profiles were of great differences, i.e., the profiles were imbalanced, which may lead to the unstable performance of the classifier on different categories. Therefore, the synthetic minority oversampling technique (SMOTE) algorithm (Chawla et al., 2002) was adopted to tackle this problem. It works by linearly synthesizing new samples for minority categories using the k-nearest neighbors concept, thereby ensuring that the quantity of samples from different categories is almost equal. This study used the SMOTE program available at https://github.com/scikit-learn-contrib/imbalanced-learn and executed it with default parameters.

### 2.6 Classification algorithms

As mentioned above, the IFS method needs a classification algorithm. For wide tests, three classification algorithms: k-nearest neighbors (kNN) (Cover and Hart, 1967), RF (Breiman, 2001), and decision tree (DT) (Safavian and Landgrebe, 1991), were attempted. These algorithms were widely used to tackling various medical problems (Chen et al., 2021; Chen et al., 2022; Ding et al., 2022; Li et al., 2022; Ran et al., 2022; Tang and Chen, 2022; Wu and Chen, 2022; Zhou et al., 2022; Wu and Chen, 2023).

#### 2.6.1 kNN

This algorithm is one of the most classic classification algorithms in machine learning. For a test sample, kNN calculates its distance to all training samples and finds *k* nearest training samples. According to the classes of these training samples, the class of the test sample is determined. Generally, the majority voting is adopted to make the decision.

#### 2.6.2 RF

RF is a classic algorithm in ensemble learning that first resamples *N* subsets from the original dataset based on the bagging strategy and uses each subset to train a decision tree classifier. Each tree is constructed by randomly selecting features. For a test sample, each tree gives its prediction. RF integrates these predictions with majority voting. Compared with decision trees, RF is more accurate and has a high generalization capability.

#### 2.6.3 DT

This algorithm is quite different from kNN and RF. Although above two algorithms can provide high performance, their principles are hard to be understood. In this regard, DT has its special merits. The classification procedures of DT are completely open. In this case, it is possible for us to understand its classification principle. Besides the tree form, DT can also be represented by a set of if-then rules, each of which contains a group of conditions and one result. The conditions may indicate a special pattern for the result, giving insights to understand essential differences of various categories.

In this study, all three abovementioned algorithms were implemented *via* the scikit-learn library. These programs were performed by using their default parameters.

### 2.7 Performance measurement

For multi-class classification, overall accuracy is the most widely used measurement. It is defined as the proportion of corrected predicted samples among all samples. However, such measurement is not perfect when the dataset is imbalanced. In this case, Mathews Correlation Coefficient (MCC) (Matthews, 1975; Jurman et al., 2012; Liu et al., 2021; Pan et al., 2022; Wang and Chen, 2022; Yang and Chen, 2022) is more accurate to evaluate the performance of classifiers. It can be computed by

$$MCC=\frac{cov\left(X,Y\right)}{\sqrt{cov\left(X,X\right)cov\left(Y,Y\right)}}$$ (1)

where *X* indicates the binary matrix of the true classes of all samples, *Y* represents the binary matrix of the predicted classes of all samples, and *cov*(.) denotes the correlation between two matrices.

Besides, F1 score was used to evaluate the performance of classifiers on each category in this study. The F1 score for one category can be computed by

$$F1\;score=\frac{2\times TP}{2\times TP+FN+FP}$$ (2)

where TP, FN and FP stand for the true positive, false negative and false positive of such category. In detail, TP is the number of accurately predicted samples in this category, FN is the number of wrongly predicted samples in this category and FP is the number of samples that belong to other categories but are predicted to be in this category. The F1 scores on all categories can be integrated to give an overall evaluation on classifiers’ performance. Generally, there are two forms to make integrations. The first one is the direct mean of F1 scores on all categories. Such measurement is called macro F1. The second one further considers the weights of categories, i.e., the weighted mean of F1 scores on all categories. It is called weighted F1.

As different measurements can induce different results, a major measurement should be determined in advance. Here, we selected weighted F1 as the major measurement.

### 2.8 Biological function enrichment

Through the above computational analysis, some important genes can be discovered from the profiles on each subtype of CD8^+^ T cells. To uncover the biological meanings behind these genes, the gene ontology (GO) and KEGG enrichment analysis was employed. The clusterProfiler 4.0 tool (Wu et al., 2021) was adopted to conduct the enrichment analysis. The threshold on *p*-value was set to 0.05 for selecting enriched GO terms and KEGG pathways.

## 3 Results

**FIGURE 1:**
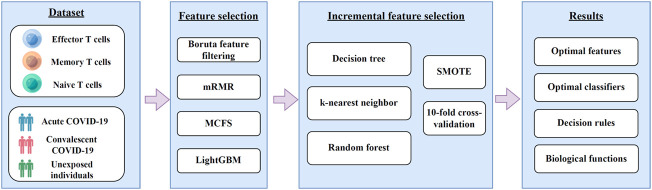
A diagram of the computational framework used in this study. We first analyzed the 3 T cell expression profiles of COVID-19 by different feature selection methods in machine learning. Then, we used the incremental feature selection method to determine the optimal features, build the optimal classifiers, and extract the important classification rules. The critical features obtained were enriched by GO and KEGG analysis to uncover their biological implications.

In this study, we first downloaded COVID-19 expression profiles for three CD8^+^ T cells subtypes, including effector, memory, and naïve T cells from GEO. Irrelevant features in the dataset on each CD8^+^ T cells subtype were excluded using Boruta, and the retained features were ranked by using mRMR, MCFS, and LightGBM in three feature ranking lists. These feature lists were then used to identify the optimal features and extract classification rules using the IFS method. The entire computational framework is shown in Figure 1.

### 3.1 Results of feature selection on CD8^+^ T cells expression profiles

For the expression profiles on each CD8^+^ T cells subtype, the Boruta method was first adopted to remove irrelevant features. 252 features remained for effector T cells. For memory and naïve T cells, 241 and 153 features were kept, respectively. These selected features were analyzed by mRMR, MCFS, and LightGBM, respectively, resulting in three feature lists for each CD8^+^ T cells subtype. These lists are provided in Supplementary Tables S1–S3.

### 3.2 Recognition of key features to distinguish COVID-19 severity on CD8^+^ T cells with the IFS method

**FIGURE 2:**
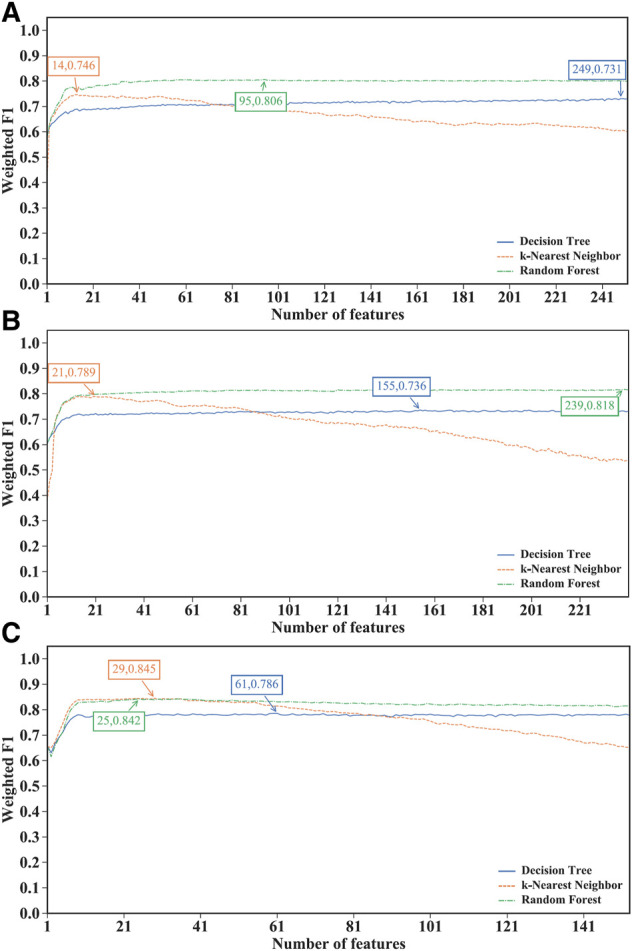
Performance of different classification algorithms with different number of features under the mRMR feature lists. **(A)** effector T cells, **(B)** memory T cells, **(C)** naïve T cells. Random forest provided the best performance on effector and memory T cells, whereas k-nearest neighbor yielded the best performance on naïve T cells.

**FIGURE 3:**
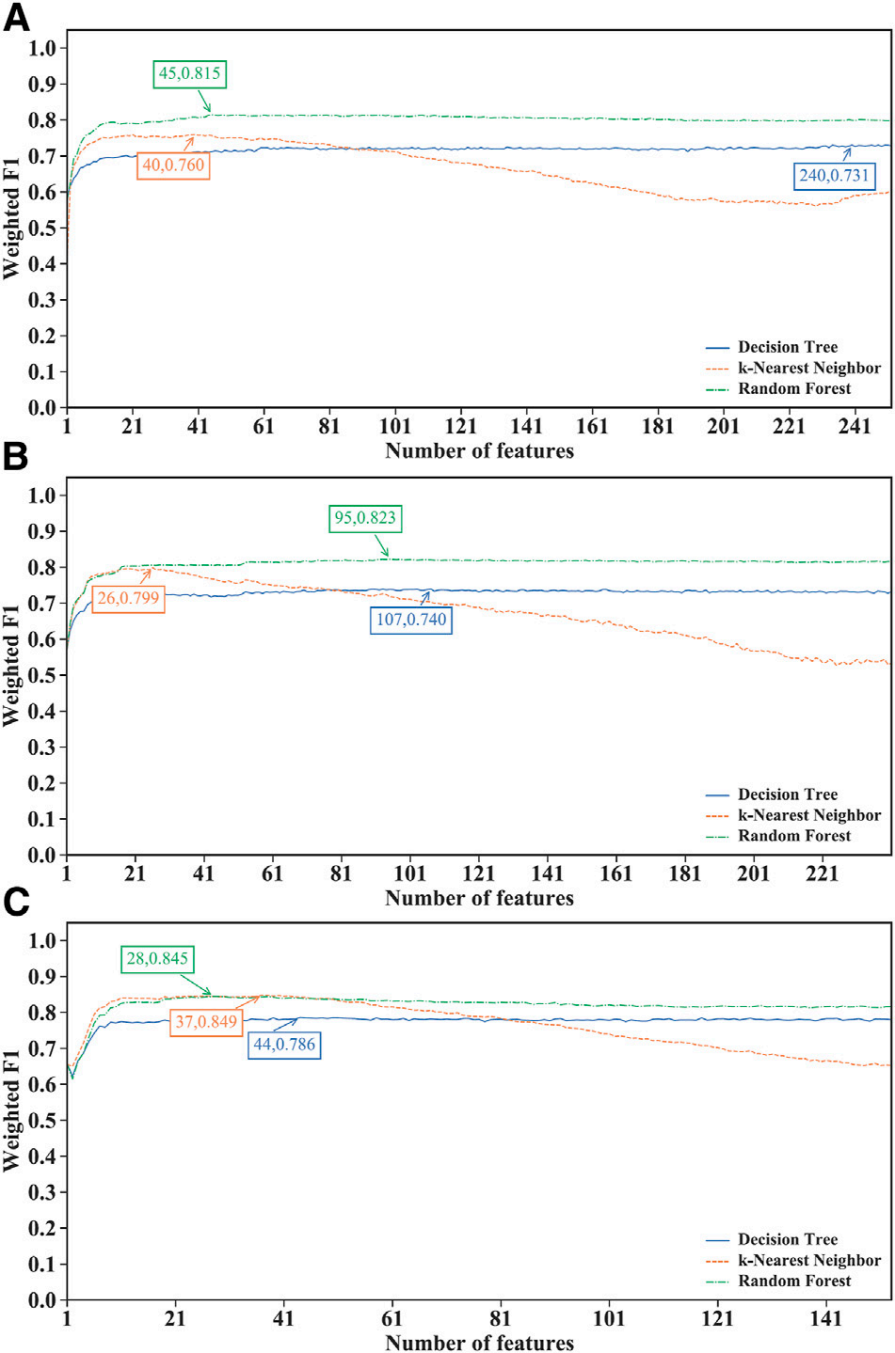
Performance of different classification algorithms with different number of features under the MCFS feature lists. **(A)** effector T cells, **(B)** memory T cells, **(C)** naïve T cells. Random forest provided the best performance on effector and memory T cells, whereas k-nearest neighbor yielded the best performance on naïve T cells.

**FIGURE 4:**
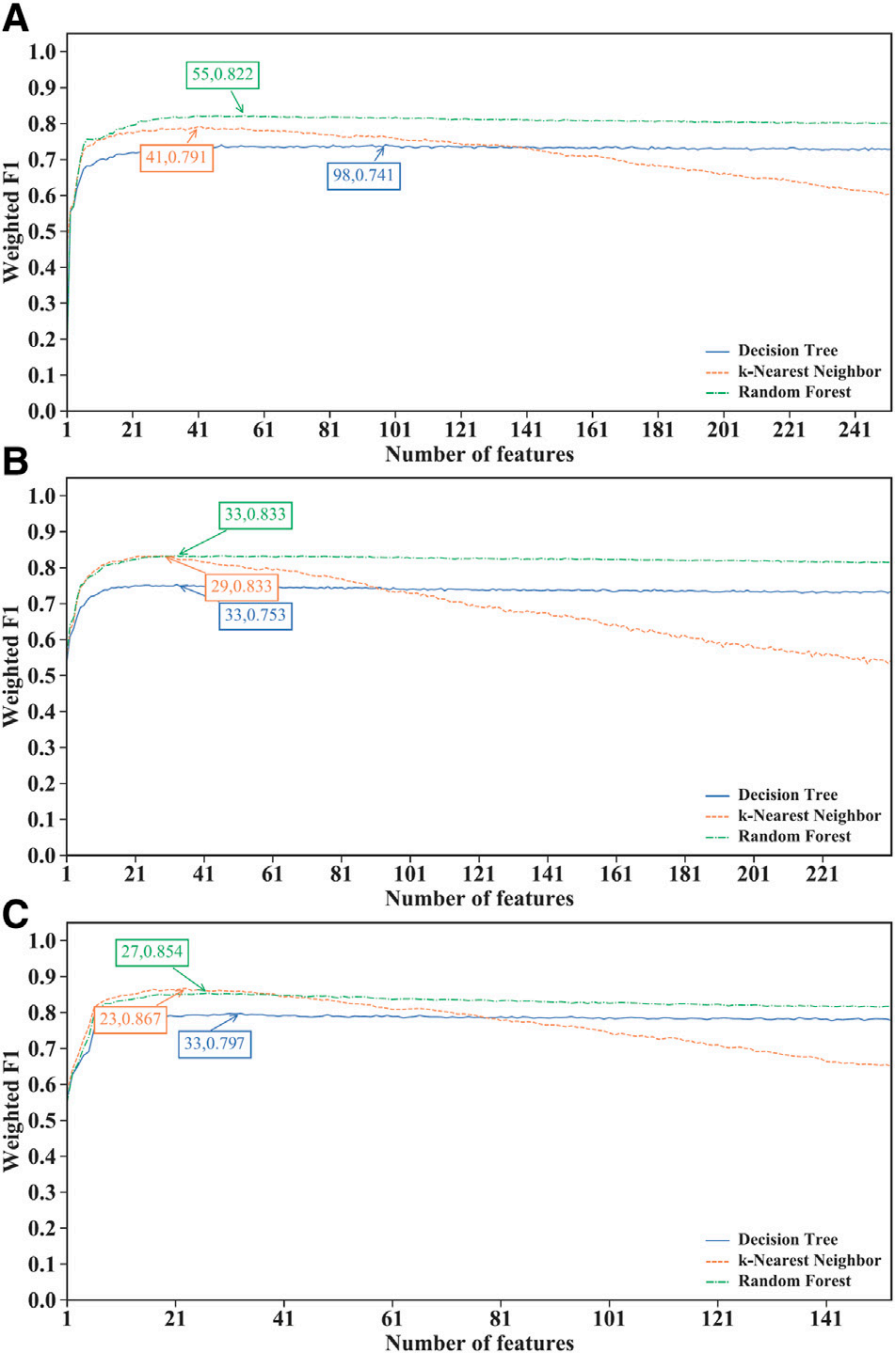
Performance of different classification algorithms with different number of features under the LightGBM feature list. **(A)** effector T cells, **(B)** memory T cells, **(C)** naïve T cells. Random forest provided the best performance on effector and memory T cells, whereas k-nearest neighbor yielded the best performance on naïve T cells.

Through the above step, three feature lists (mRMR, MCFS and LightGBM feature lists) were obtained for each CD8^+^ T cells subtype. However, important features for the classification task are still difficult to determine. Therefore, the IFS method was used to find the optimal features and construct the optimal classifiers, which constructed a series of classifiers and calculated their performance metrics. The IFS results for the three CD8^+^ T cells subtypes using different feature lists are provided in Supplementary Tables S4–S6. The IFS curves were plotted to observe the trend of the classifiers’ performance, measured by weighted F1, under the changing of feature numbers, as shown in Figures 2–4.

For the IFS results on the mRMR feature lists of three CD8^+^ T cells subtypes (Supplementary Table S4), the IFS curves are shown in Figure 2. For the effector T cells, DT, kNN and RF reached the highest performance when the first 249, 14 and 95 features were used with weighted F1 values of 0.731, 0.746 and 0.806 (Figure 2A). For the memory T cells, three classification algorithms yielded the maximum weighted F1 values of 0.736, 0.789 and 0.818 when first 155, 21 and 239 features were adopted (Figure 2B). As for the naïve T cells, the highest weighted F1 values for three classification algorithms were 0.786, 0.845, and 0.842 (Figure 2C), which were obtained by using top 61, 29 and 25 features in the list. Clearly, for effector and memory T cells, RF provided better performance than DT and kNN, whereas kNN was best for the naïve T cells. Accordingly, we can construct the optimal RF classifiers for effector and memory T cells, and the optimal kNN classifier for the naïve T cells based on the mRMR feature lists. The overall performance of the above optimal classifiers, measured by ACC, MCC and macro F1, is listed in Table 2. ACC and MCC values were all no less than 0.8 and 0.67, respectively, indicating the good performance of these classifiers.

**TABLE 2:** Detailed performance of the optimal classifiers obtained by using the mRMR, MCFS, and LightGBM methods for three cell subtypes.

Cell subtype	Feature ranking method	Classification algorithm	Number of features	ACC	MCC	Macro F1	Weighted F1
Effector T cells	mRMR	RF	95	0.809	0.670	0.748	0.806
	MCFS	RF	45	0.815	0.688	0.776	0.815
	LightGBM	RF	55	0.823	0.698	0.787	0.822
Memory T cells	mRMR	RF	239	0.821	0.694	0.786	0.818
	MCFS	RF	95	0.824	0.704	0.798	0.823
	LightGBM	RF	33	0.834	0.724	0.815	0.833
Naïve T cells	mRMR	kNN	29	0.841	0.755	0.826	0.845
	MCFS	kNN	37	0.844	0.761	0.828	0.849
	LightGBM	kNN	23	0.863	0.787	0.847	0.867

Of the IFS results on the MCFS feature lists of three CD8^+^ T cells subtypes (Supplementary Table S5), Figure 3 shows the IFS curves. For the effector T cells, RF achieved the highest weighted F1 of 0.815 using the first 45 features (Figure 3A). Other two classification algorithms provided the highest weighted F1 values of 0.731 and 0.760 when top 240 and 40 features were adopted. For the memory T cells, the IFS curves of three classification algorithms reached the highest points with the top 107, 26 and 95 features with weighted F1 values of 0.740, 0.799 and 0.823 (Figure 3B). For the naïve T cells, kNN obtained the highest weighted F1 of 0.849 using the first 37 features (Figure 3C). DT and RF yielded the highest weighted F1 values of 0.786 and 0.845 when top 44 and 28 features were used. It was interesting that the performance of three classification algorithms on the MCFS feature lists was similar to that on the mRMR feature lists. RF was best on effector and memory T cells, whereas kNN was best on the naïve T cells. Likewise, three optimal classifiers can be built on three CD8^+^ T cells subtypes based on the MCFS feature lists. Their detailed overall performance is also listed in Table 2. ACC and MCC values were all higher than 0.81 and 0.68, respectively, suggesting high performance of these classifiers.

For the IFS results on the LightGBM feature lists of three CD8^+^ T cells subtypes (Supplementary Table S6), IFS curves are illustrated in Figure 4. For the effector T cells, DT/kNN/RF achieved the maximum weighted F1 of 0.741/0.791/0.822 when the first 98/41/55 features were used (Figure 4A). For the memory T cells, DT/kNN/RF peaked at 33/29/33 features with a weighted F1 value of 0.753/0.833/0.833 (Figure 4B). For the naïve T cells, DT/kNN/RF gained the maximum weighted F1 value of 0.797/0.867/0.854 when the first 33/23/27 features were used (Figure 4C). It was surprising that RF was still better than DT and kNN on effector and memory T cells, and kNN was still better than DT and RF on the naïve T cells, similar to the results on mRMR and MCFS feature lists. This also increased the reliability of our results. Likewise, three optimal classifiers on three CD8^+^ T cells subtypes can be set up based on the LightGBM feature lists. Table 2 lists the detailed overall performance of these classifiers. ACC and MCC values were all higher than 0.82 and 0.69, respectively, indicating their high performance.

**FIGURE 5:**
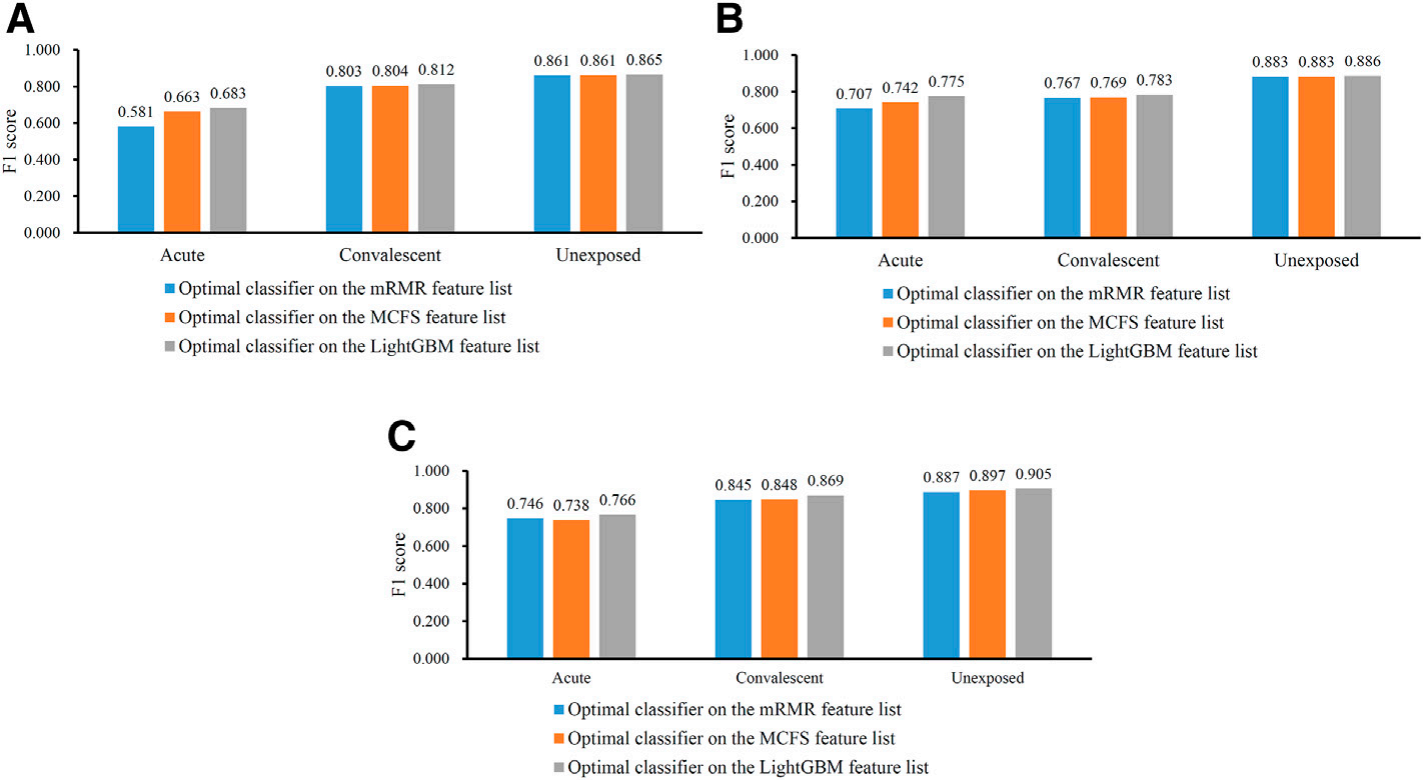
Performance of the optimal classifiers for three CD8^+^ T cells subtypes on three categories. **(A)** effector T cells, **(B)** memory T cells, **(C)** naïve T cells. The optimal classifiers on the LightGBM feature lists were better than those on other two feature lists.

In Table 2, the overall performance of nine optimal classifiers on different feature lists and cell subtypes is provided. We further extracted their performance on three categories (acute, convalescent and unexposed), measured by F1 score, which are shown in Figure 5. It can be observed that on each cell subtype, optimal classifier on the LightGBM feature list always provided the highest performance on all categories, generally followed by the optimal classifiers on the MCFS and mRMR feature lists. Such results also conformed to their overall performance (Table 2). Furthermore, all classifiers generally yielded best performance on unexposed individuals, followed by convalescent and acute COVID-19.

**FIGURE 6:**
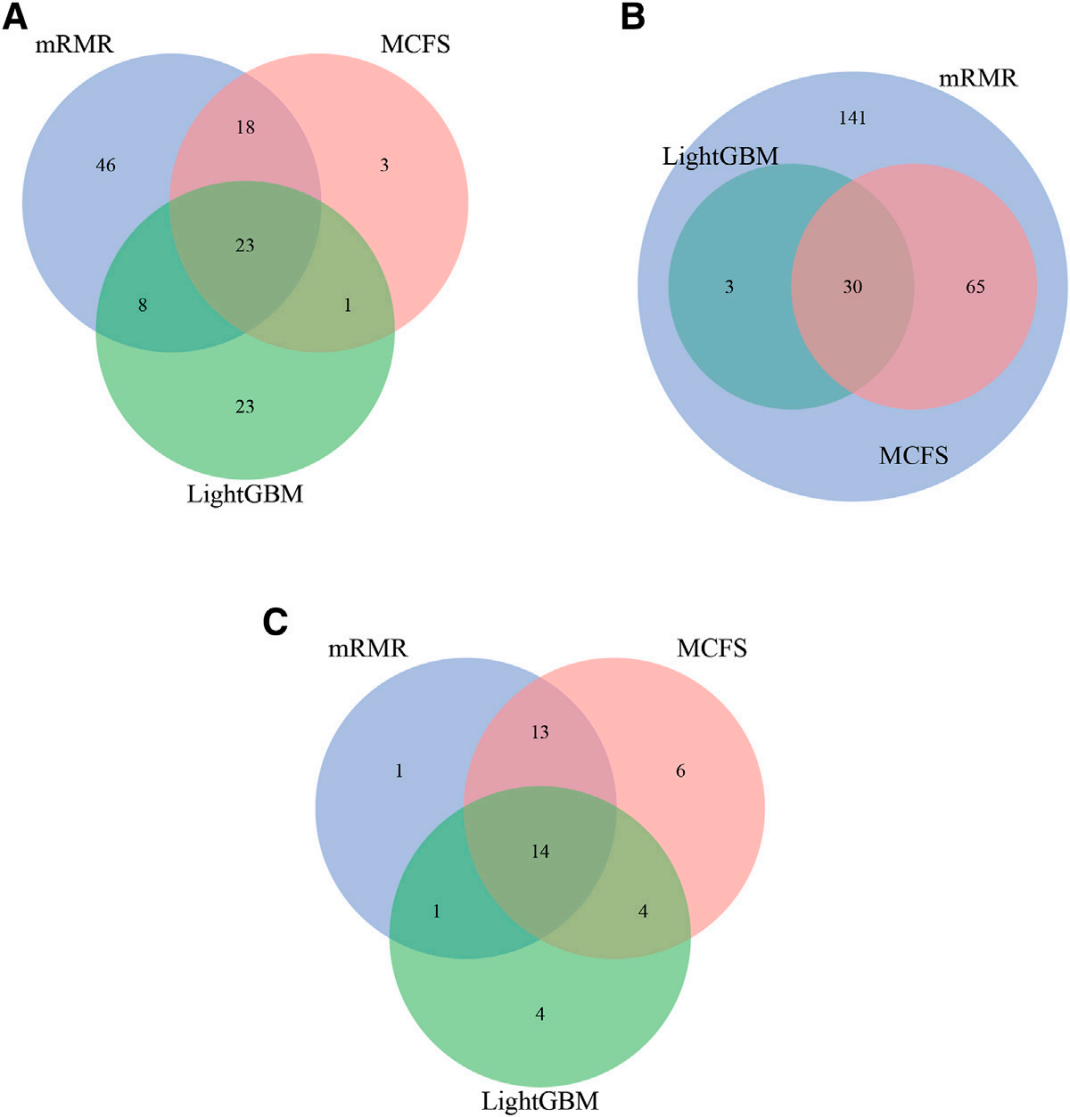
Intersection results of the optimal feature sets based on different feature lists yielded by three feature ranking methods for three CD8^+^ T cells subtypes. **(A)** effector T cells, **(B)** memory T cells, **(C)** naïve T cells.

For each CD8^+^ T cells subtype, three optimal classifiers were constructed based on three feature lists. The features used in these classifiers (i.e., optimal features) can be obtained, comprising three optimal feature sets. It is interesting to investigate the intersection of these three optimal feature sets using Venn diagrams. The Venn diagrams are provided in Figure 6. The detailed intersection results are shown in Supplementary Table S7. It can be observed that there were 23 important features in three optimal feature sets for effector T cells (Figure 5A). For the memory T cells, 30 important features were included in three optimal feature sets (Figure 5B). The three optimal feature subsets under the naïve T cells had 14 essential features intersected (Figure 5C). The biological mechanisms of these important feature genes are described in the Section 4.

### 3.3 Classification rules for important features in the CD8^+^ T cells profiles

**FIGURE 7:**
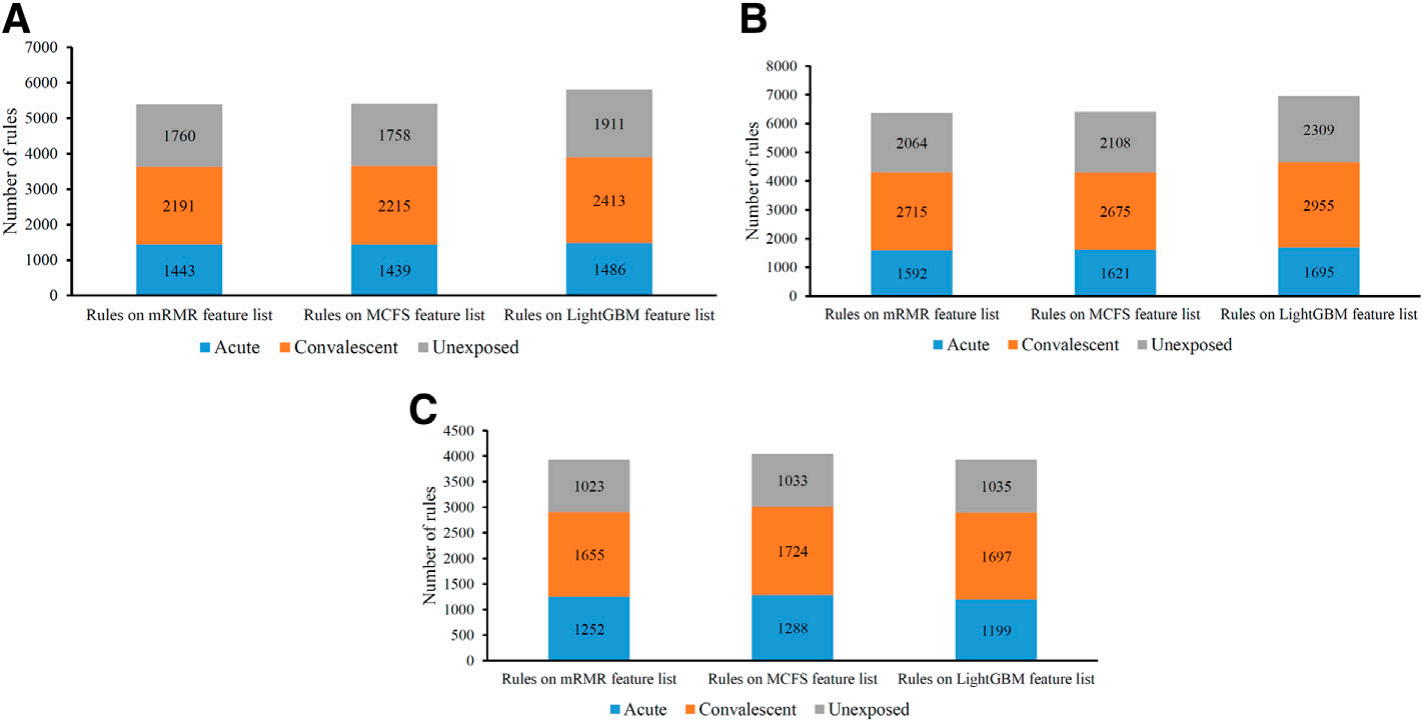
Distribution of rules yielded by decision trees on three categories in three CD8^+^ T cells subtypes. **(A)** effector T cells, **(B)** memory T cells, **(C)** naïve T cells.

On each CD8^+^ T cells subtype, DT always provided the lowest performance under a given feature list. The performance is listed in Table 3. However, it has the special merit that is not shared by kNN and RF. From the constructed DT, several classification rules can be obtained, which implies the special patterns on each category. Thus, we further employed DT to investigate profiles on three CD8^+^ T cells subtypes. As mentioned in Section 3.2, the optimal features for DT can be found by executing IFS method on different feature lists of three CD8^+^ T cells subtypes. With these optimal features, DT was applied on all samples to learn a large tree, from which a group of classification rules were obtained. These classification rules on three CD8^+^ T cells subtypes and three feature lists are shown in Supplementary Table S8. The number of rules on each CD8^+^ T cells subtype and feature list is listed in Table 3. A fair number of rules were obtained, which provides informative reference for revealing the relationships between expression patterns of key feature genes and three categories. For each rule set, some rules were for acute COVID-19, whereas others were for convalescent COVID-19 or unexposed individuals. The number of rules for each category is illustrated in Figure 7. It can be observed that convalescent COVID-19 was always assigned most rules, whereas the rules on unexposed individuals were the second most on effector and memory T cells, and acute COVID-19 was assigned the second most rules on Naïve T cells.

**TABLE 3:** Details of the optimal DT classifiers obtained for each cell subtype under different feature ranking methods and the number of rules extracted.

Cell subtype	Feature ranking method	Number of features	Weighted F1	Number of rules
Effector T cells	mRMR	249	0.731	5394
	MCFS	240	0.731	5412
	LightGBM	98	0.741	5810
Memory T cells	mRMR	155	0.736	6371
	MCFS	107	0.740	6404
	LightGBM	33	0.753	6959
Naïve T cells	mRMR	61	0.786	3930
	MCFS	44	0.786	4045
	LightGBM	33	0.797	3931

### 3.4 Immune functions for genes identified in the optimal feature sets

**FIGURE 8:**
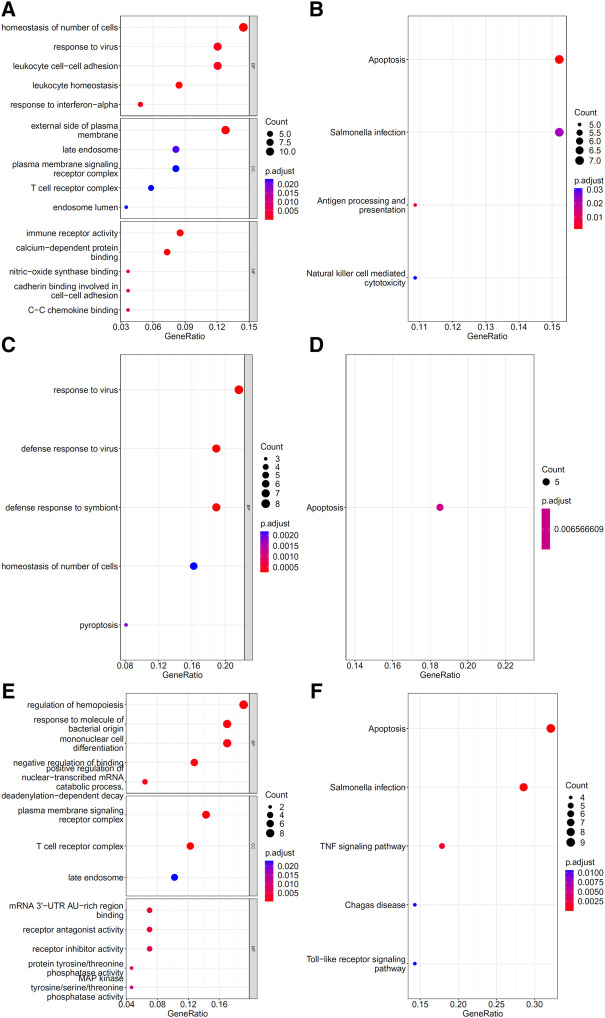
Results of the functional enrichment analysis on the optimal genes for different feature lists in the effector T cells. Top GO terms (**(A)**: mRMR, **(C)**: MCFS, and **(E)**: LightGBM) and KEGG pathways (**(B)**: mRMR, **(D)**: MCFS, **(F)**: LightGBM) are shown.

**FIGURE 9:**
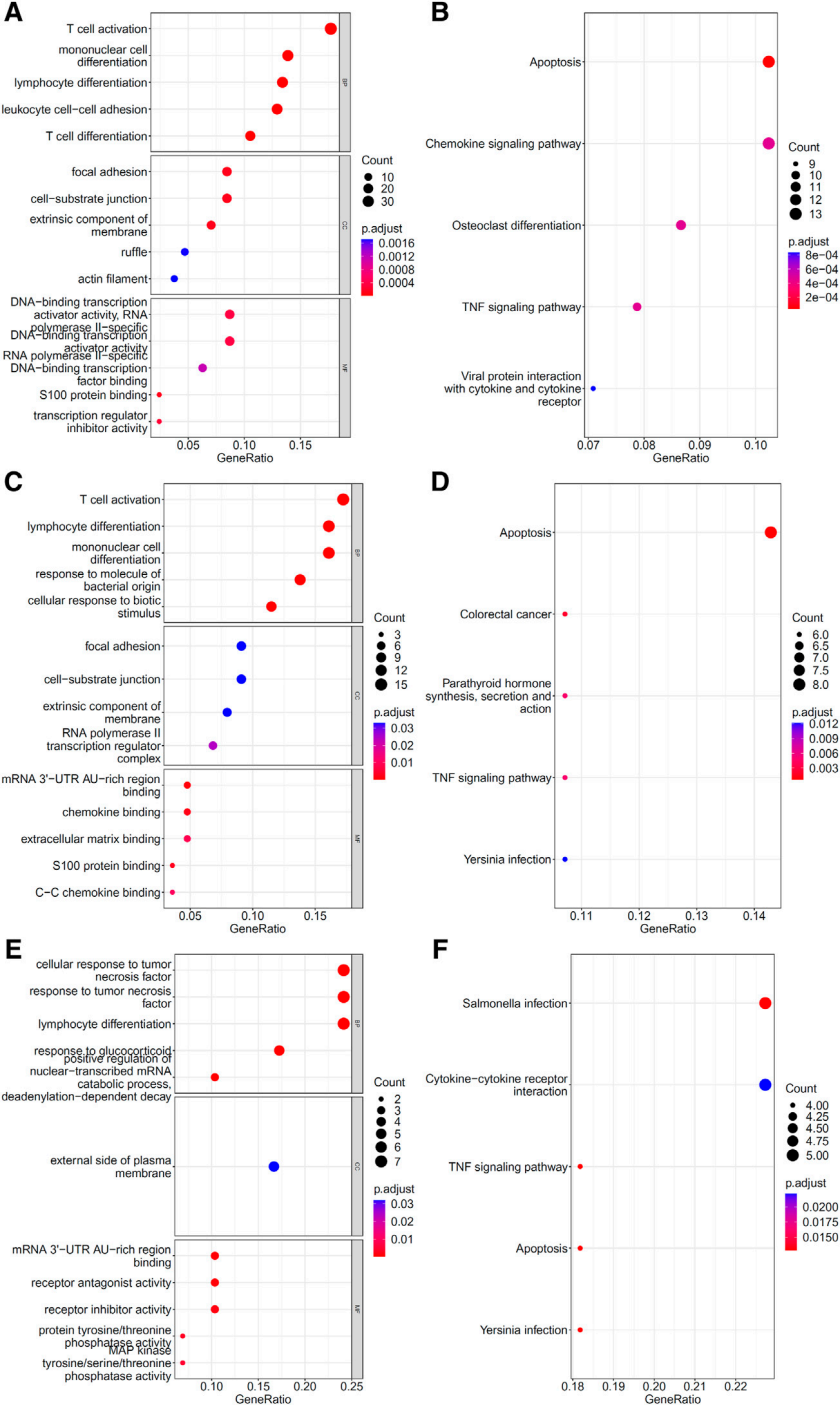
Results of the functional enrichment analysis on the optimal genes for the different feature lists in the memory T cells. Top GO terms (**(A)**: mRMR, **(C)**: MCFS, and **(E)**: LightGBM) and KEGG pathways (**(B)**: mRMR, **(D)**: MCFS, **(F)**: lightGBM) are shown.

**FIGURE 10:**
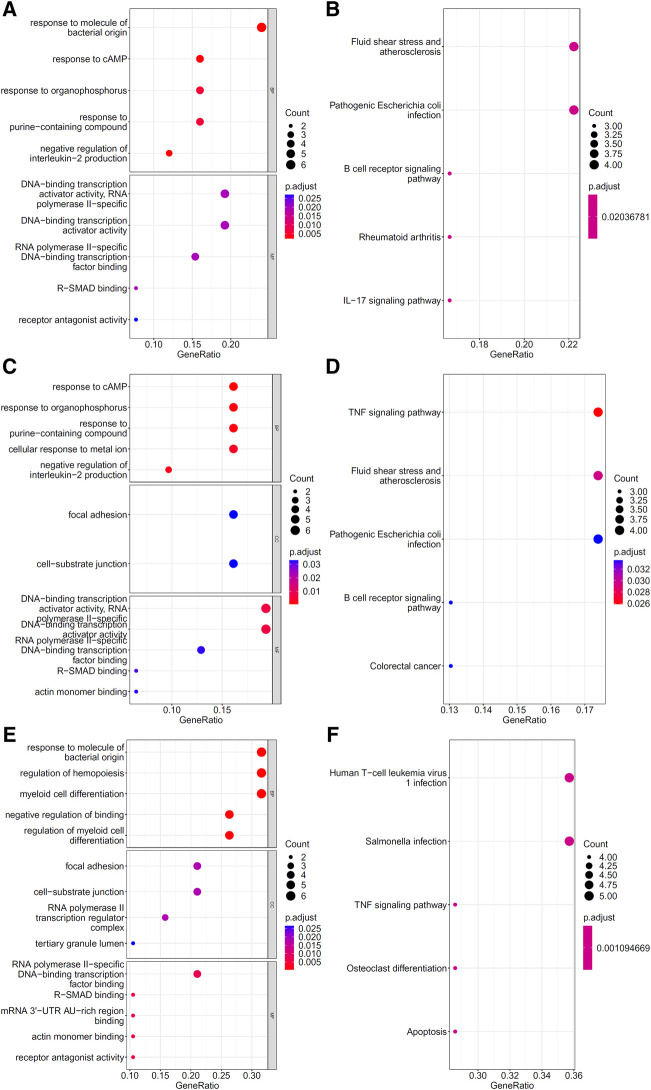
Results of the functional enrichment analysis on the optimal genes for the different feature lists in the naïve T cells. Top GO terms (**(A)**: mRMR, **(C)**: MCFS, and **(E)**: LightGBM) and KEGG pathways (**(B)**: mRMR, **(D)**: MCFS, **(F)**: lightGBM) are shown.

To explore the biological functions and pathways involved in the essential genes for each CD8^+^ T cells subtype, we performed GO and KEGG enrichment analyses on the genes in the optimal feature sets obtained under each feature ranking list for each subtype of CD8^+^ T cells. The results are provided in Supplementary Tables S9–S11. The top five GO terms and KEGG pathways from the enrichment results are shown in Figures 8–10. For the effector T cells, the main biological functions enriched are response to virus, homeostasis of number of cells, T cell receptor complex, and signaling pathways, including apoptosis and *salmonella* infection (Figure 8). For the memory T cells, the enrichment results contain T cell activation, lymphocyte differentiation, mononuclear cell differentiation, and signaling pathways, such as apoptosis and TNF signaling pathway (Figure 9). For the naïve T cells, the enrichment results were for GO terms, such as response to cAMP, response to organophosphorus, and signaling pathways, e.g., B-cell receptor signaling pathway and TNF signaling pathway (Figure 10). These critical biological functions and signaling pathways are developed in the Section 4.

## 4 Discussion

For each CD8^+^ T cell subtype, we obtained three sets of features that are important to distinguish the disease state of patients with COVID-19 through three feature ranking algorithms and IFS method. Next, we conducted GO and KEGG enrichment analyses for all the genes in the three groups of features to facilitate our interpretation of these key genes. We discussed genes to confirm their important roles in COVID-19 according to existing studies. The main discussion results of each cell subtype were organized as follows.

### 4.1 Functional analysis of the key features of CD8^+^ effector T cells

The effector CD8^+^ T cells that respond to antigen stimulation proliferate and differentiate. Some will eventually differentiate into memory CD8^+^ T cells. Among the features that can distinguish CD8^+^ Effector T cells in different stages of COVID-19 infection, we found that they mainly contain cytotoxic genes (GZMA, GZMK, and PRF1), T cell receptor (TCR)-related genes (TRBV4.2 and TRBV7.2), cytokine-related genes (IFITM2, IL7R, and IL32), and others. At the same time, our functional enrichment results also showed the relationship between these genes and immune killing, as follows: GO:0009615 (response to virus), GO:0042101 (T cell receptor complex), GO:0140375 (immune receptor activity), and hsa04210 (apoptosis).

CD8^+^ effector T cells are essential for adaptive immunity against COVID-19 virus infection, and the cytotoxic response intensity of CD8^+^ Effector T cells also corresponds to different stages of antiviral immunity. Patients with COVID-19 infection have higher levels of GZMs and PRF1 than healthy controls; they also have characteristic expression changes during infection recovery (Wen et al., 2020; Westmeier et al., 2020). During viral infection, TCRs recombine to generate a functional and highly diverse TCR repertoire crucial for CD8 effector T cells to identify and kill infected cells (Luo et al., 2021). Therefore, the dynamic changes of genes, such as TRBV4.2, TRBV7.2 (TCR components), and IL7R (associated with V(D)J recombination), may be related to different periods of infection. Other genes, such as FITM2, reportedly restrict the entry of COVID-19 virus into cells (Winstone et al., 2021), but its expression in CD8^+^ Effector T cells and its dynamic characteristics at different stages of infection have not been studied.

### 4.2 Functional analysis of the key features of CD8^+^ memory T cells

There are two types of CD8^+^ memory T cells, namely, effector memory T cells (Tem) and central memory T cells (Tcm). CD8^+^ Tcm mainly reside in secondary lymphoid organs and can rapidly be converted into effector cells upon antigen stimulation, whereas CD8^+^ Tem is mainly distributed in peripheral tissues and can respond rapidly to stimulation by producing effector cytokines. In different stages of infection, CD8^+^ Memory T cells have different activation, proliferation, and secretion states (Tavukcuoglu et al., 2021). GO/KEGG enrichment analysis for the features in our results also revealed that many genes were associated with T cell differentiation and effector activity, such as: GO:0042110 (T cell activation), GO:0030217 (T cell differentiation), hsa04062 (chemokine signaling pathway), and hsa04061 (viral protein interaction with cytokine and cytokine receptor). In these genes, features associated with cellular activation and differentiation (B2M, IL7R, ZFP36, ZFP36L1, ZFP36L2, CD8A, KLF6, and LGALS1) may be related to the function of CD8^+^ memory T cells at different stages of infection, whereas features associated with cell chemotaxis (SELL, CCL5, CXCR4, and NFKBIA) could be linked to the recruitment of CD8^+^ memory cells (Xiong et al., 2020). The COVID-19 virus may also escape the immune system through chemokines (Khalil et al., 2021), suggesting that the expression of chemokines may be associated with the different stages of infection.

### 4.3 Functional analysis of the key features of CD8^+^ naïve T cells

The GO enrichment results of key genes show that they are related to the response to multiple stimuli, such as: GO:0071216 (cellular response to biotic stimulus) and GO:0051591 (response to cAMP). cAMP has been shown to play an important role in the initial activation and effector differentiation of naïve CD8^+^ T cells (Linnemann et al., 2009). At different stages of infection in COVID-19 patients, naïve CD8^+^ T cells responded to different levels of antigenic stimulation (Wen et al., 2020; Fenoglio et al., 2021), which resulted in different proportions and activation states of naïve CD8^+^ T cells; this phenomenon may help distinguish different disease states. In addition, a correlation was found between the proportion of naive CD8^+^ T cells and infection severity (Moderbacher et al., 2020).

At the gene expression level, some genes showed important roles in differentiating infection stages and were identified by all three feature ranking algorithms. Among these genes, the protein product of the ZFP36 gene belongs to the zinc finger family and has been linked to the regulation of gene expression and cellular response to growth factor stimulation. Studies on COVID-19 showed that ZFP36 inhibited T cell activation, and proliferation during viral infection and the expression level of ZFP36 changed dramatically during infection (Xiong et al., 2020). DUSP1 was downregulated in COVID-19 infection and may be associated with enhanced MAPK pathway activation and steroid resistance (Sharif-Askari et al., 2021). Our features also contained some inflammatory genes (FOS, JUN, and KLF6), which may be related to the inflammatory state at different disease stages; these genes have different expression levels during COVID-19 infection and recovery (Wen et al., 2020).

## 5 Conclusion

In this study, the single-cell RNA-Seq datasets under three subtypes of CD8^+^ T cells (effector, memory, and naïve T cells) related to COVID-19 infection, convalescent, and unexposed were deeply investigated. Several advanced computational methods were applied on these datasets. Essential genes, interpretable classification rules and efficient classifiers were obtained. The former two results can deepen our understanding on the mechanism of the regulatory role of CD8^+^ T cells on COVID-19. The last one can be useful tools to distinguish patients’ COVID-19 severity in terms of CD8^+^ T cells.

## Data Availability

Publicly available datasets were analyzed in this study. This data can be found here: https://www.ncbi.nlm.nih.gov/geo/query/acc.cgi?acc=GSE188429.

## References

[B1] Araf Y. Akter F. Tang Y. D. Fatemi R. Parvez M. S. A. Zheng C. (2022). Omicron variant of SARS-CoV-2: Genomics, transmissibility, and responses to current COVID-19 vaccines. J. Med. Virol. 94, 1825–1832. 10.1002/jmv.27588 35023191 PMC9015557

[B2] Breiman L. (2001). Random forests. Mach. Learn. 45, 5–32. 10.1023/a:1010933404324

[B3] Chawla N. V. Bowyer K. W. Hall L. O. Kegelmeyer W. P. (2002). SMOTE: Synthetic minority over-sampling technique. J. Artif. Intell. Res. 16, 321–357. 10.1613/jair.953

[B4] Chen G. Wu D. Guo W. Cao Y. Huang D. Wang H. (2020). Clinical and immunological features of severe and moderate coronavirus disease 2019. J. Clin. Invest. 130, 2620–2629. 10.1172/JCI137244 32217835 PMC7190990

[B5] Chen W. Chen L. Dai Q. (2021). iMPT-FDNPL: identification of membrane protein types with functional domains and a natural language processing approach. Comput. Math. Methods Med. 2021, 7681497. 10.1155/2021/7681497 34671418 PMC8523280

[B6] Chen L. Li Z. Zhang S. Zhang Y.-H. Huang T. Cai Y.-D. (2022). Predicting RNA 5-methylcytosine sites by using essential sequence features and distributions. Biomed. Res. Int. 2022, 4035462. 10.1155/2022/4035462 35071593 PMC8776474

[B7] Cover T. Hart P. (1967). Nearest neighbor pattern classification. IEEE Trans. Inf. Theory 13, 21–27. 10.1109/tit.1967.1053964

[B8] Dan J. M. Mateus J. Kato Y. Hastie K. M. Yu E. D. Faliti C. E. (2021). Immunological memory to SARS-CoV-2 assessed for up to 8 months after infection. Science 371, eabf4063. 10.1126/science.abf4063 33408181 PMC7919858

[B9] Ding S. Wang D. Zhou X. Chen L. Feng K. Xu X. (2022). Predicting heart cell types by using transcriptome profiles and a machine learning method. Life 12, 228. 10.3390/life12020228 35207515 PMC8877019

[B10] Draminski M. Rada-Iglesias A. Enroth S. Wadelius C. Koronacki J. Komorowski J. (2008). Monte Carlo feature selection for supervised classification. Bioinformatics 24, 110–117. 10.1093/bioinformatics/btm486 18048398

[B11] Fenoglio D. Dentone C. Parodi A. Di Biagio A. Bozzano F. Vena A. (2021). Characterization of T lymphocytes in severe COVID‐19 patients. J. Med. Virol. 93, 5608–5613. 10.1002/jmv.27037 33913544 PMC8242373

[B12] Fiolet T. Kherabi Y. Macdonald C. J. Ghosn J. Peiffer-Smadja N. (2022). Comparing COVID-19 vaccines for their characteristics, efficacy and effectiveness against SARS-CoV-2 and variants of concern: a narrative review. Clin. Microbiol. Infect. 28, 202–221. 10.1016/j.cmi.2021.10.005 34715347 PMC8548286

[B13] Francis J. M. Leistritz-Edwards D. Dunn A. Tarr C. Lehman J. Dempsey C. (2022). Allelic variation in class I HLA determines CD8(+) T cell repertoire shape and cross-reactive memory responses to SARS-CoV-2. Sci. Immunol. 7, eabk3070. 10.1126/sciimmunol.abk3070 34793243 PMC9017864

[B14] Gong J. Zhan H. Liang Y. He Q. Cui D. (2021). Role of Th22 cells in human viral diseases. Front. Med. 8, 708140. 10.3389/fmed.2021.708140 PMC8381044 34434945

[B15] Grifoni A. Weiskopf D. Ramirez S. I. Mateus J. Dan J. M. Moderbacher C. R. (2020). Targets of T Cell responses to SARS-CoV-2 coronavirus in humans with COVID-19 disease and unexposed individuals. Cell 181, 1489–1501. 10.1016/j.cell.2020.05.015 32473127 PMC7237901

[B16] Jurman G. Riccadonna S. Furlanello C. (2012). A comparison of MCC and CEN error measures in multi-class prediction. PLoS ONE 7, e41882. 10.1371/journal.pone.0041882 22905111 PMC3414515

[B17] Ke G. Meng Q. Finley T. Wang T. Chen W. Ma W. (2017). “LightGBM: A highly efficient gradient boosting decision tree,” in 31st Conference on Neural Information Processing Systems (NIPS 2017), Long Beach, CA, USA, December 4–9, 2017

[B18] Khalil B. A. Elemam N. M. Maghazachi A. A. (2021). Chemokines and chemokine receptors during COVID-19 infection. Comput. Struct. Biotechnol. J. 19, 976–988. 10.1016/j.csbj.2021.01.034 33558827 PMC7859556

[B19] Kohavi R. (1995). “A study of cross-validation and bootstrap for accuracy estimation and model selection,” in International joint Conference on artificial intelligence, Montreal, QC, August 20–25, 1995 (Lawrence Erlbaum Associates), 1137–1145.

[B20] Kotturi M. F. Peters B. Buendia-Laysa F. Jr. Sidney J. Oseroff C. Botten J. (2007). The CD8+ T-cell response to lymphocytic choriomeningitis virus involves the L antigen: uncovering new tricks for an old virus. J. Virol. 81, 4928–4940. 10.1128/JVI.02632-06 17329346 PMC1900207

[B21] Kursa M. B. Rudnicki W. R. (2010). Feature selection with the Boruta package. J. Stat. Softw. 36, 1–13. 10.18637/jss.v036.i11

[B22] Li X. Lu L. Chen L. (2022). Identification of protein functions in mouse with a label space partition method. Math. Biosci. Eng. 19, 3820–3842. 10.3934/mbe.2022176 35341276

[B23] Linnemann C. Schildberg F. A. Schurich A. Diehl L. Hegenbarth S. I. Endl E. (2009). Adenosine regulates CD8 T‐cell priming by inhibition of membrane‐proximal T‐cell receptor signalling. Immunology 128, e728–e737. 10.1111/j.1365-2567.2009.03075.x 19740334 PMC2753927

[B24] Liu H. A. Setiono R. (1998). Incremental feature selection. Appl. Intell. 9, 217–230. 10.1023/a:1008363719778

[B25] Liu H. Hu B. Chen L. Lu L. (2021). Identifying protein subcellular location with embedding features learned from networks. Curr. Proteomics 18, 646–660. 10.2174/15701646mtex2nzc51

[B26] Luo L. Liang W. Pang J. Xu G. Chen Y. Guo X. (2021). Dynamics of TCR repertoire and T cell function in COVID-19 convalescent individuals. Cell Discov. 7, 89. 10.1038/s41421-021-00321-x 34580278 PMC8476510

[B27] Matthews B. (1975). Comparison of the predicted and observed secondary structure of T4 phage lysozyme. Biochim. Biophys. Acta 405, 442–451. 10.1016/0005-2795(75)90109-9 1180967

[B28] Moderbacher C. R. Ramirez S. I. Dan J. M. Grifoni A. Hastie K. M. Weiskopf D. (2020). Antigen-specific adaptive immunity to SARS-CoV-2 in acute COVID-19 and associations with age and disease severity. Cell 183, 996–1012. 10.1016/j.cell.2020.09.038 33010815 PMC7494270

[B29] Nguyen Q. P. Deng T. Z. Witherden D. A. Goldrath A. W. (2019). Origins of CD4(+) circulating and tissue-resident memory T-cells. Immunology 157, 3–12. 10.1111/imm.13059 30897205 PMC6459775

[B30] Pan X. Chen L. Liu I. Niu Z. Huang T. Cai Y. D. (2022). Identifying protein subcellular locations with embeddings-based node2loc. IEEE/ACM Trans. Comput. Biol. Bioinform. 19, 666–675. 10.1109/TCBB.2021.3080386 33989156

[B31] Peng H. Long F. Ding C. (2005). Feature selection based on mutual information: criteria of max-dependency, max-relevance, and min-redundancy. IEEE Trans. Pattern Anal. Mach. Intell. 27, 1226–1238. 10.1109/TPAMI.2005.159 16119262

[B32] Ran B. Chen L. Li M. Han Y. Dai Q. (2022). Drug-Drug interactions prediction using fingerprint only. Comput. Math. Methods Med. 2022, 7818480. 10.1155/2022/7818480 35586666 PMC9110191

[B33] Safavian S. R. Landgrebe D. (1991). A survey of decision tree classifier methodology. IEEE Trans. Syst. Man. Cybern. 21, 660–674. 10.1109/21.97458

[B34] Samudrala P. K. Kumar P. Choudhary K. Thakur N. Wadekar G. S. Dayaramani R. (2020). Virology, pathogenesis, diagnosis and in-line treatment of COVID-19. Eur. J. Pharmacol. 883, 173375. 10.1016/j.ejphar.2020.173375 32682788 PMC7366121

[B35] Sanyal S. (2020). How SARS-CoV-2 (COVID-19) spreads within infected hosts - what we know so far. Emerg. Top. Life Sci. 4, 371–378. 10.1042/ETLS20200165 33269805 PMC7733667

[B36] Sharif-Askari F. S. Sharif-Askari N. S. Goel S. Hafezi S. Assiri R. Al-Muhsen S. (2021). SARS-CoV-2 attenuates corticosteroid sensitivity by suppressing DUSP1 expression and activating p38 MAPK pathway. Eur. J. Pharmacol. 908, 174374. 10.1016/j.ejphar.2021.174374 34303662 PMC8295491

[B37] Slifka M. K. Whitton J. L. (2000). Antigen-specific regulation of T cell-mediated cytokine production. Immunity 12, 451–457. 10.1016/s1074-7613(00)80197-1 10843378

[B38] Tang S. Chen L. (2022). iATC-NFMLP: Identifying classes of anatomical therapeutic chemicals based on drug networks, fingerprints and multilayer perceptron. Curr. Bioinform. 17, 814–824. 10.2174/1574893617666220318093000

[B39] Tavukcuoglu E. Horzum U. Inkaya A. C. Unal S. Esendagli G. (2021). Functional responsiveness of memory T cells from COVID-19 patients. Cell. Immunol. 365, 104363. 10.1016/j.cellimm.2021.104363 33905951 PMC8052500

[B40] Wang R. Chen L. (2022). Identification of human protein subcellular location with multiple networks. Curr. Proteomics 19, 344–356. 10.2174/1570164619666220531113704

[B41] Wen W. Su W. Tang H. Le W. Zhang X. Zheng Y. (2020). Erratum: Author Correction: Immune cell profiling of COVID-19 patients in the recovery stage by single-cell sequencing. Cell Discov. 6, 41. 10.1038/s41421-020-00187-5 32595980 PMC7305225

[B42] Westmeier J. Paniskaki K. Karaköse Z. Werner T. Sutter K. Dolff S. (2020). Impaired cytotoxic CD8+ T cell response in elderly COVID-19 patients. MBio 11, e02243–e02220. 10.1128/mBio.02243-20 32948688 PMC7502863

[B43] Winstone H. Lista M. J. Reid A. C. Bouton C. Pickering S. Galao R. P. (2021). The polybasic cleavage site in SARS-CoV-2 spike modulates viral sensitivity to type I interferon and IFITM2. J. Virol. 95, e02422–e02420. 10.1128/JVI.02422-20 33563656 PMC8104117

[B44] Wu Z. Chen L. (2022). Similarity-based method with multiple-feature sampling for predicting drug side effects. Comput. Math. Methods Med. 2022, 9547317. 10.1155/2022/9547317 35401786 PMC8993545

[B45] Wu C. Chen L. (2023). A model with deep analysis on a large drug network for drug classification. Math. Biosci. Eng. 20, 383–401. 10.3934/mbe.2023018 36650771

[B46] Wu T. Hu E. Xu S. Chen M. Guo P. Dai Z. (2021). clusterProfiler 4.0: A universal enrichment tool for interpreting omics data. Innovation. 2, 100141. 10.1016/j.xinn.2021.100141 34557778 PMC8454663

[B47] Xiong Q. Peng C. Yan X. Yan X. Chen L. Sun B. (2020). Characteristics of SARS-CoV-2-specific cytotoxic T cells revealed by single-cell immune profiling of longitudinal COVID-19 blood samples. Signal Transduct. Target. Ther. 5, 285. 10.1038/s41392-020-00425-y 33277469 PMC7716113

[B48] Yang Y. Chen L. (2022). Identification of drug–disease associations by using multiple drug and disease networks. Curr. Bioinform. 17, 48–59. 10.2174/1574893616666210825115406

[B49] Zheng H. Y. Zhang M. Yang C. X. Zhang N. Wang X. C. Yang X. P. (2020). Elevated exhaustion levels and reduced functional diversity of T cells in peripheral blood may predict severe progression in COVID-19 patients. Cell. Mol. Immunol. 17, 541–543. 10.1038/s41423-020-0401-3 32203186 PMC7091621

[B50] Zhou X. Ding S. Wang D. Chen L. Feng K. Huang T. (2022). Identification of cell markers and their expression patterns in skin based on single-cell RNA-sequencing profiles. Life 12, 550. 10.3390/life12040550 35455041 PMC9025372

